# Emerging alternative model for cardiothoracic surgery training in India

**DOI:** 10.3402/meo.v18i0.20961

**Published:** 2013-05-06

**Authors:** Rajan Vaithianathan, Senthil Panneerselvam

**Affiliations:** Mahatma Gandhi Medical College and Research Institute, Pondicherry, India

**Keywords:** thoracic surgery, training, India

## Abstract

**Background:**

In India, cardiothoracic (CT) surgery training follows a 3+3-year model, where 3 years of general surgery residency with certification (MS/DNB) is required for entering 3 years of thoracic surgery residency (MCh/DNB). There are two certifying boards at the national level. One being the Medical Council of India (MCI), which oversees the major accreditation process involving the undergraduate and postgraduate medical education in India, and the other being the National Board of Examinations (NBE), which was formed for the purpose of establishing a uniform standard of postgraduate medical education. Recently, the latter body has come up with an alternative model for thoracic surgery residency in India. This model includes an integrated 6-year residency, with lesser emphasis on general surgical skills and greater exposure to CT surgery.

**Conclusions:**

Changes to the current model of training for CT surgery is the need of the hour and should be initiated very soon by the MCI to meet the future demand for CT surgeons in India. An integrated training program is essential to create a new generation of cardiovascular specialists. Future directions to achieve this goal must include modifications to the undergraduate programs so as to infuse interest for CT surgery in the young minds of medical students.

## Introduction

With a teeming population of more than 1.1 billion people, the Indian health-care machinery is struggling to cope up with the rising burden due to both communicable and chronic diseases. Many studies on Indian immigrants and cross-sectional studies from India have shown a high prevalence of coronary artery disease (CAD) in the Indian population and this figure is several times higher than in industrialized nations (1). Moreover, there exists an additional burden due to the coexisting 5 million people with rheumatic heart diseases. Nearly 40% of these two groups require cardiac surgery and added to these numbers are about 1.5 million children requiring surgical correction for congenital heart defects. There are about 50 cardiothoracic (CT) training programs, with 100 residents completing training every year. The total number of CT surgeons in India is only about 1,000, performing 70–80,000 surgeries per year in 174 centers (2). Hence, there is an urgent necessity to clear the huge backlog in CT procedures as well as to plan for the increasing demand in the future. There is a strong need for more centers across the country to impart an efficient and attractive CT training program to encourage young surgeons to take up this demanding surgical super specialty.

## Burden of CT problems in India

An estimate from the extrapolated results of Global Burden of Diseases study done in 2000 showed that around 30 million patients may be suffering from CAD in India (1). Concrete data is not available about the incidence of congenital heart diseases, but approximately 200,000 babies are born every year with some form of congenital CT defect. With the aging population, degenerative diseases of the aorta are also increasing and there is a strong likelihood that the burden of cardiovascular disabilities will increase in the future. Nearly 50,000 cases of carcinoma lung and 20,000 cases of esophageal cancers are diagnosed annually in India. Approximately 60,000 open-heart surgeries are performed in India annually, and most of these are for coronary artery and valvular heart disease. Almost 5,000 surgeries are being performed for congenital heart diseases alone (3).

## Historical perspective of CT surgery training in India

The first thoracic surgery residency program in India was established in the year 1958 by Dr. Reeve H. Betts at Christian Medical College and Hospital (CMC) in Vellore, a city in south India (4). Initially, the duration of training was for 2 years and in due course, it has been increased to 3 years. A candidate has to complete 3 years of residency in general surgery before entering next 3 years of thoracic surgery training. In this model, the skills gained during general surgery residency are mostly related to surgical pathologies involving the breast, thyroid gland, gastrointestinal tract, head and neck, and other benign disorders, such as hernia and hydrocele. The maximum exposure to thoracic surgery is only for a period of 1–2 months and this takes place during the sub-specialty rotations in the 2nd year of residency. Later, the candidate has to enter 3 years of CT program (MCh), after completing an entrance exam. Thus, this model entails 6 arduous years of training, more in the form of apprenticeship, leaving very little scope for the young thoracic surgeon to practice independently on completing training. They work under a senior CT surgeon for some years to refine his/her skills in performing complicated CT procedures. This model has been in existence for more than 50 years in the Indian system of surgical training. No major changes have taken place in the course requirements, curriculum, and the duration of the general surgical training or the CT training.

## Current status of CT surgery training in India

On completing surgical residency, the prospective candidates have the options of joining either an MCh (Master of Chirurgiae) program, accredited by the Medical Council of India (MCI), or the DNB (diplomate of national board) program, accredited by the National Board of Examinations (NBE). The MCI is the primary organization for the accreditation process of undergraduate and postgraduate medical training in India. The NBE, set up in 1975, runs a parallel system of postgraduate education, with uniform selection criteria, curriculum, and examination format for training positions offered all over India (5). All training programs run by the NBE are recognized by the MCI and vice versa.

The current status of CT surgery training in India has suffered a setback due to the decreasing number of applicants and increasing number of vacant training slots. Reports suggest that this speciality is not a popular choice among surgical residents (3, 6). The major reasons that have contributed to this situation are:

1. Decreasing open-heart surgeries due to the advent of interventional cardiology.
2. Decreasing job market.
3. Lack of confidence in performing surgeries independently, without supervision, at the end of residency.
4. Majority of residents want to settle early in life, have a job with better incentives and security.

With the decrease in applications and unfilled vacancies, a huge shortage is predicted by the year 2020 for thoracic surgeons in India. In the southern state of Tamil Nadu, which comprises one of the largest training centers in India, more than 50% of MCh CT surgery positions were unfilled for the year 2011 (7). The scenario did not improve in the year 2012, again 30% of positions remained vacant and candidates failed to achieve a minimum score required for getting admission in the speciality (8). Again, this is due to fewer applicants for thoracic surgery and pooling of more applications from eligible candidates for the specialities of urology, surgical oncology, and surgical gastroenterology. The situation is no different even in the institute that started the first thoracic surgery residency in India. For the year 2012, in CMC, Vellore, where the first training program was established in India, a second round of examination and interviews had to be held for the unfilled training positions in thoracic surgery (9). The situation is more or less similar in the training slots offered through the NBE. This is an alarming state of affairs, remembering the ever-increasing cardiac disorders in the Indian population and the increasing thoracic visceral malignancies diagnosed every year. This scenario is very much similar to that in the United States, where there has been a progressive fall in the number of applications received for thoracic surgery training.

## Recent model for CT training in the United States

In the Unites States, the conservative model for training is 5+2 years, wherein a 5-year residency in general surgery is required to enter 2 years of fellowship in cardiac surgery. Studies show a 4.4% decrease in the number of applications for thoracic surgery training slots from 1994 to 2002. From 2004, it worsened with less applications than the number of training slots, with a fill rate of 72% (10). On the other hand, available data predicts a 46% increase in demand for thoracic surgeons by the year 2025 in the United States, owing to an increase in population and aging (11). This led to the introduction of an integrated 6-year thoracic surgery residency. Eligibility criteria include: completion of medical school, approved internship, and a pass in medical licensing examinations. Until 2012, implementation of the new model has taken place only in a limited number of programs, around 17 in number, with a total of 20 training positions. However, all vacancies were filled in the 2012 NRMP match. The applicant-to-vacancy position ratio was 4:1, with a total of 80 applicants for every 20 slots (12). On the other hand, for the 2-year fellowship model, for 102 vacancy positions, only 90 certified applicants enrolled in the NRMP match, with a final fill rate of 76% (13). Thus, the integrated residency model has met with more success than the 2-year fellowship model in the United States.

A report from the Medical College of Wisconsin had shown promising results, with a significant increase in the number of applications for the 6-year integrated thoracic surgery training program. Additionally, the 6-year applicants appeared to be more academically accomplished than previous applicants to the traditional 2-year program. While it is still early days, it appears that interest in thoracic surgery is high among medical students and the institution of a 6-year program has the potential to once again attract the ‘best and the brightest’ to this specialty (14).

## Emerging model for CT training program in India

The NBE introduced a training format in 2011, analogous to the integrated thoracic surgery residency in the United States. The total duration of training is 6 years, with examinations at the end of the 2nd and 5th year of training. Conducting the exit exam in 5th year would allow the candidates to fully dedicate and concentrate on improving their surgical skills in other advanced CT procedures. The final year of residency has rotations in the sub-specialties of pediatric cardiac surgery, robotic cardiac surgery, and video-assisted thoracic surgery at other institutes of excellence. In the description about the format, the curriculum states ‘… training includes reasonable exposure to general surgery and critical care management, pertinent with cardio vascular and thoracic surgery’. Training has generally been divided into parts 1 and 2. Part 1 includes the first 2 years of residency. Training is imparted on basic skills in general surgery, applied basic sciences pertinent to cardiovascular and thoracic surgery, cardiovascular engineering, critical care medicine, diagnostic imaging, and allied specialties, such as cardiology. Training in 2nd to 5th year of residency (part 2) is primarily in cardio vascular surgical departments, with rotations in allied specialties, such as cardiac anesthesia, pulmonary medicine, and critical care unit. A surgical log book has to be maintained and a minimum number of procedures as provided in the curriculum have to be performed either independently or under supervision. Research methodology is part of the training similar to other Indian postgraduate courses. A candidate is required to present a dissertation, publish a paper in an indexed journal, and make two paper presentations at a national or international conference in the specialty. At the end of 6 years of training, having met all of the above-mentioned criteria, a candidate is eligible to practice thoracic surgery in India.

## Pros and cons

The main advantage in this model is that the total period of residency spent in general surgery is limited to 9 months, during which core surgical skills are gained by the trainees. As mentioned earlier, this paves the way for a greater emphasis on learning skills relevant to thoracic surgery and allied specialties. An additional 6th year of rotation, in sub-specialties, such as pediatric cardiac surgery, video-assisted thoracic surgery, etc., at other institutes of excellence, provides ample opportunities for the trainees to learn advanced, state-of-the-art CT procedures. This would provide ample exposure in using these new tools to build a career as future cardiovascular specialists. Also, specific training in allied specialties, such as nuclear medicine, diagnostic radiology, cardiac anesthesia, and critical care will be an important add-on to their training, thus leading to a holistic approach to patient care. The most striking aspect of this unique integrated curriculum is having the entire residency dedicated to what the trainees will be doing for the rest of their careers, thus leading to better trained surgeons and more gratification for the trainees.

There have been notable advancements in technology in recent times and conventional open surgical treatments are giving way to less invasive strategies. Management protocols are frequently employing percutaneous devices (such as coronary artery stents, peripheral arterial stenting, endovascular stent-grafts for aneurysmal disease, and placement of transcatheter aortic valves), robotic and videoscopic/thoracoscopic/endoscopic tools for minimal access approaches. The next generation of CT surgeons must be proficient in traditional open surgical procedures as well as being at ease using these new state-of-the-art procedures, and the integrated program may be the best platform to achieve this goal.

However, longer duration of training from the point of obtaining MBBS qualification and the necessity to make an early career choice of specialization are factors that can make this training model difficult for the prospective trainees. New graduates are relatively immature when tasked with choosing their future specialty as CT surgery. The major limiting factor is lack of familiarity with CT surgery, its scope, and its challenges. To overcome this hurdle and to infuse interest for CT surgical career in these young minds, programs could be created to expose them to academic CT surgery through active mentorship, operative skills training, and individual academic pursuits. Allen et al. in their study of a cohort of 18 medical students, showed success in these pursuits, as demonstrated by the high academic productivity of their students and their 80% matriculation rate to surgical residencies (15).

## Conclusions

Unlike in the United States, where the integrated residency model was introduced 5 years earlier, it has been only 2 years since the introduction of a similar model in India. Moreover, the CT courses accredited by the MCI still follow the standard 3+3 years of residency training. A limited number of institutes accredited by the NBE still follow the standard model. In this scenario, one has to wait and see if this new training format meets with success as in the United States and whether the primary accreditation body, MCI, will try to implement the format in its programs. Modifications should be made to the undergraduate curriculum so as to stimulate interest in the CT specialty at a much earlier phase. In conclusion, to achieve its goals, this new format of training should ensure the complete and efficient transference of advanced surgical skills from the mentor to the CT trainees.
